# Gender trends in Canadian medicine and surgery: the past 30 years

**DOI:** 10.1186/s12909-024-05071-4

**Published:** 2024-01-30

**Authors:** Lauren Pickel, Nirojini Sivachandran

**Affiliations:** 1 Temerty Faculty of Medicine, University of Toronto, Toronto, Canada; 2 Toronto Retina Institute, Toronto, Canada; 3 Department of Chemistry and Biology, Toronto Metropolitan University, Toronto, ON Canada

**Keywords:** Medical education, History, Canada, Gender, Women, Surgery, Medicine

## Abstract

**Background:**

While the number of women entering medicine has steadily increased since the 1970s in Canada, the gender composition along each stage of the medical training pathway has not been comprehensively reported. We therefore sought to systematically examine the gender composition of students, residents, and practicing physicians over the past 30 years in Canada.

**Results:**

In this cross-sectional analysis of Canadian medical trainees including MD applicants (137,096 male, 169,099 female), MD students (126,422 male, 152, 967 female), MD graduates (29,413 male, 34,173 female), residents by the decade (24,425 male, 28,506 female) and practicing surgeons (total 7,457 male, 3,457 female), we find that increased female representation in medicine is not matched by representation in surgery, with the key being the specialty choice process. The likelihood of female applicants matriculating to medical school was less than male applicants in the 90s (OR 0.92, 95% CI 0.92–0.93), greater in the early 2000s (OR 1.03, 95% CI 1.03–1.04), and has since balanced out (OR 1.00, 95% CI 1.00-1.01), with medical school classes being nearly 60% female for the past two decades. Despite this, females have remained underrepresented in most surgical residency programs, with odds of female medical students entering surgical residency other than Ob/Gyn being about half that of male students (OR 0.56, 95% CI 0.44–0.71), resulting in a slow increase in practicing female surgeons of less than 0.5% per year in many surgical disciplines and projected parity decades or centuries in the future.

**Conclusions:**

While undergraduate medical education has been majority female in Canada for nearly three decades, females remain greatly underrepresented in the physician workforce within surgical specialties. To build a representative medical workforce equipped to care for diverse patient populations, factors influencing the specialty choices of early career physicians will need to be examined and addressed.

## Background

According to the most recent data from the Canadian Medical Association (CMA), 42.7% of Canada’s active physicians are female [1]. This proportion continues to rise, with graduating classes being majority female in the past 20 years. Female physicians have been demonstrated to perform well in their respective fields of practice, as measured by internists’ rates of mortality and readmission [2]; surgeons’ rates of post-operative complications, mortality, or readmission [3]; and primary care doctors’ management of cancer screening, diabetes, and prevention of hospitalization [4].

However, despite steadily increasing proportions of female students entering medical school since the 1940s, there are still gender disparities across specialties, particularly within surgery. While this trend is commonly acknowledged, the gender distribution of medical training has not been systematically examined in Canada. Understanding the evolution and current state of gender equity in medical training has implications for medical education and equitable patient care. The objective of the present study was to examine the proportion of women comprising each stage of medical training. Possible factors that may differentially influence the specialty choices of female or other historically underrepresented groups of students are discussed.

## Methods

The data range 1990 to 2020 was chosen to examine trends in the past three decades, during which time the proportion of MD degrees awarded to men and women by Canadian universities equalized. Data on awarded MD degrees were also available from 1940 and included. Data past the 2020/2021 year was not yet available from all sources at the time of collection. Data sources captured male and female as gender, and we have therefore retained this terminology. The number and gender of applicants, successful matriculants, and graduates from all MD programs across Canada was retrieved from the Association of Faculties of Medicine of Canada (AFMC) Canadian Medical Education Statistics for each year beginning in 1990-91. Applicant gender was not reported by University of Toronto beginning in 2014, and by any Ontario university beginning in 2019. Residency training data was obtained from the Canadian Post-M.D. Education Registry (CAPER) annual census. Subcategories were included within the larger specialty (e.g., Gynecologic Oncology within Obstetrics/Gynecology; Colorectal surgery within General Surgery; Thoracic surgery within Cardiac and Thoracic Surgery). Data on currently practicing physicians were obtained from the Canadian Institute for Health Information (CIHI). Data were compiled in Excel. Linear regression and odds ratio calculations were performed in R.

## Results

**Fig. 1:**
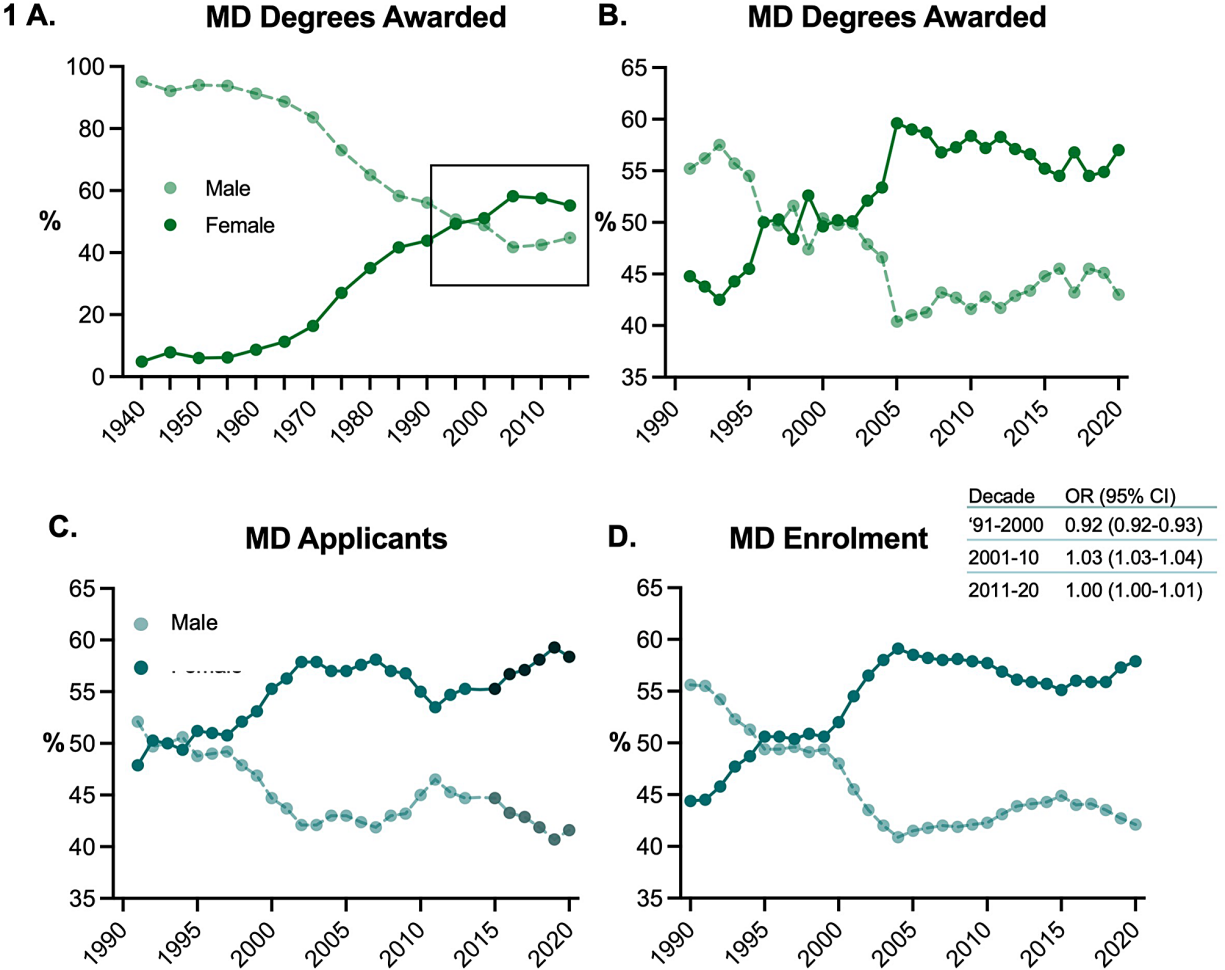
Proportion of female graduating medical doctors every 5 years since 1940 (**A**) and yearly since 1990 (**B**), demonstrating convergence in 1996. (**C**) Proportion of female applicants to Canadian MD programs. Note MD applicant gender was not reported by some Ontario universities beginning in 2014. (**D**) Proportion female first year MD program students. Odds ratio (OR) and 95% confidence interval (CI) indicate relative likelihood of female vs. male applicants matriculating, pooled by decade

This cross-sectional analysis included 306,195 MD applicants; 279,389 MD students; 63,588 graduating MDs; 13,834 residents in family medicine, internal medicine, and surgical disciplines by decade; and 10,914 practicing surgeons in Canada between 1990 and 2020. Data on MD degrees are available from nearly a century ago, when fewer than 5% of graduating MDs were women (Fig. 1A). This gradually began to shift in the middle of the 20th century, and the graduating class of 1996 was the first cohort to be 50% female (Fig. 1B). Since 2005, graduating classes have hovered between 55 and 60% female. Parallel trends are seen in the history of MD applicants and enrolment. The relative odds of matriculation to a Canadian MD program as a female student were lower in the 1990’s (OR 0.92, 95% CI 0.92–0.93) and higher in the 2000’s (OR 1.03, 95% CI 1.03–1.04), then appear to have equalized in the 2010’s, though notably not all schools reported applicant gender after 2013 (Fig. 1C, D). The age distribution of applicants and matriculants is similar between males and females (data not shown). In the past two admissions cycles (2019/2020 and 2020/2021), entering students have been 58.2% and 59% female, respectively.

The numbers of students entering different specialties continues to vary significantly by gender. Looking at first year residents in large specialty categories, female students are more likely to enter family medicine than male students (60% female in 2020, Fig. 2A). Medical specialties and surgical specialties have become approximately even in recent years, having 51.3% and 49.9% female in 2020, respectively. However, amongst surgical specialities, this is in large part driven Ob/Gyn (85.8% female), and when surgical specialties apart from Ob/Gyn were considered, these residency programs were 39.8% female.

**Fig. 2:**
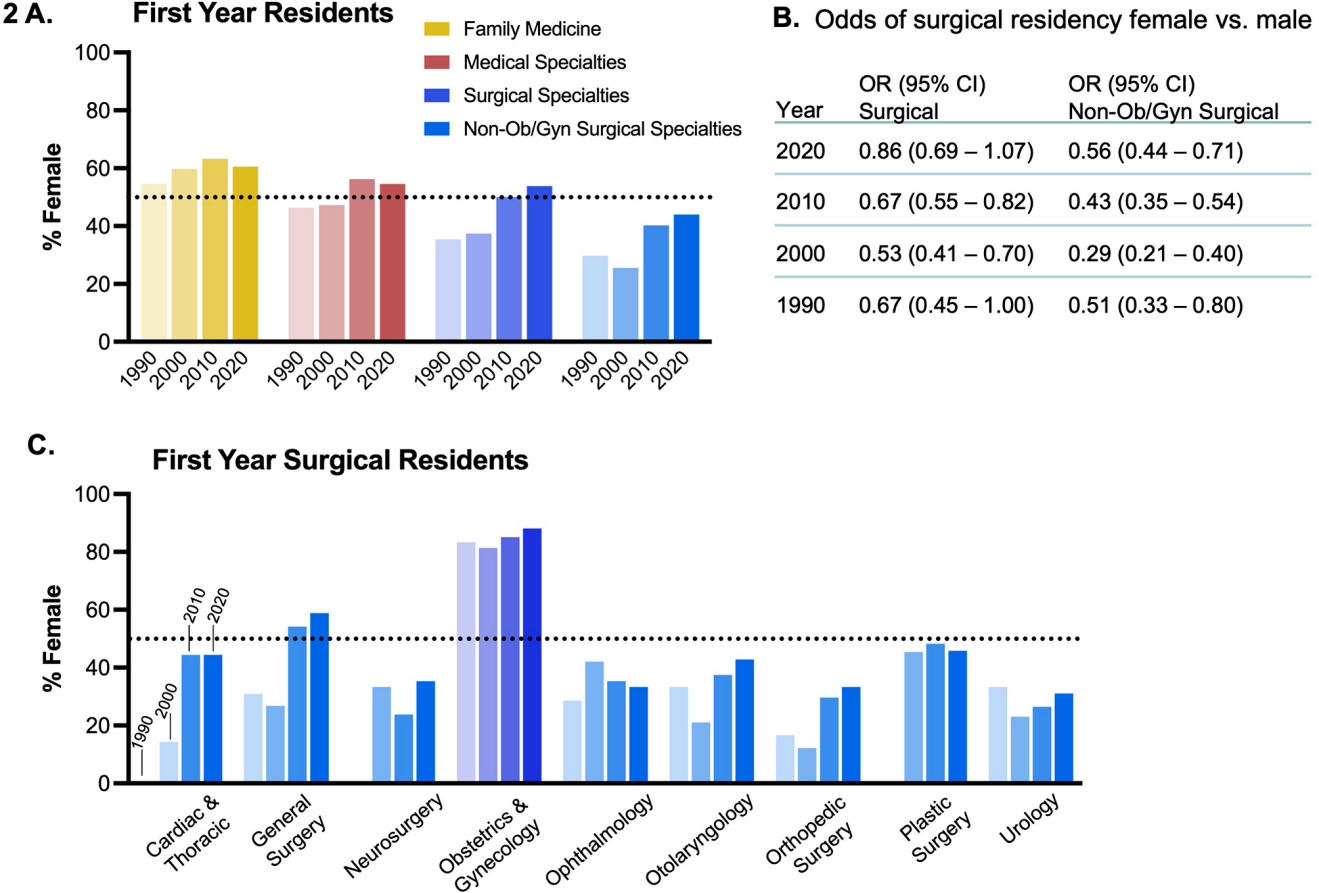
(**A**) Percent female first-year residents in non-surgical and surgical specialties. (**B**) Odds of female vs. male graduating medical students entering first-year residency in a surgical discipline. (C) Percent female first year residents in each surgical specialty. Grouped bars represent years 1990, 2000, 2010, and 2020 respectively for each specialty or group of specialties. Data from CAPER.

Considering the relative odds of female compared to male medical students entering surgery in the context of a changing medical school class gender composition, female students were significantly less likely to enter any surgical discipline until the most recent decade (Fig. 2B). When considering surgical disciplines apart from Ob/Gyn, the odds of female students entering surgery are about half that of male students, even in the most recent decade. Within individual surgical subspecialties (Fig. 2C), female residents make up the largest proportion of first-year residents in Ob/Gyn (88.1%). In the past two decades, general surgery has also had an increase in female first year residents (from 26.8% in 2000 to 58.8% in 2020), and plastic surgery is approaching parity. On the other hand, specialties such as neurosurgery, ophthalmology, orthopedic surgery, and urology remain at around one third female representation.

**Fig. 3:**
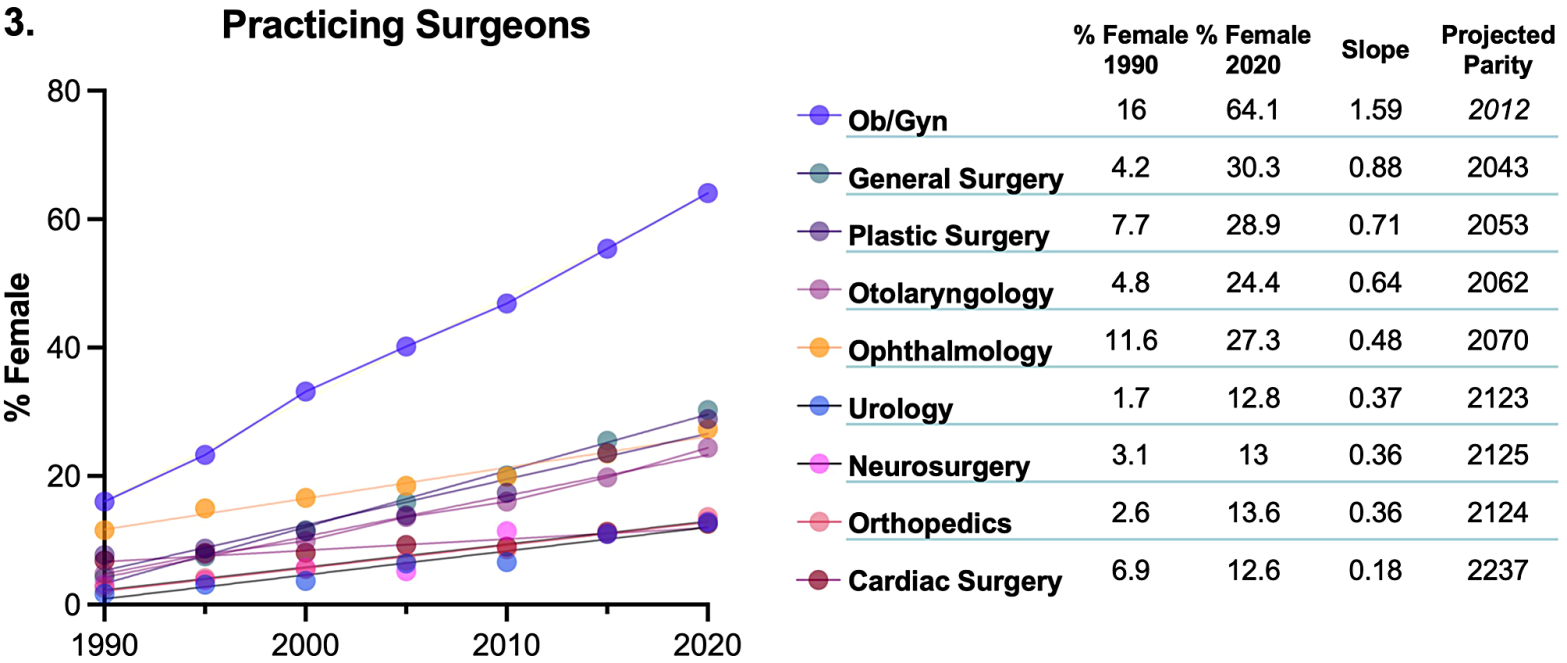
Percent female practicing physicians across surgical subspecialties, rate of increase in female representation since 1990, and projected year of gender parity based on this trend. Data from CIHI.

Reflecting the trends in residency training, the number of practicing female surgeons has gradually risen over the past three decades across all specialties (Fig. 3). The greatest rate of increase was in obstetrics and gynecology (1.59% per year increase), which was followed by general surgery (0.88% per year), plastic surgery (0.71%), and otolaryngology (0.64%). The rate of increase in female representation was less than half a percent per year in ophthalmology (0.48%), urology (0.37%), orthopedic surgery and neurosurgery (both 0.36%), and the increase in practicing female surgeons has been the slowest in cardiac surgery (0.18% per year). To illustrate these trends, the predicted years that gender parity would be achieved at the current rates in each specialty are given. While general surgery and plastic surgery would see gender parity in practicing surgeons before the next 30-year update, otolaryngology and ophthalmology will lag by an additional one to two decades. It would be a century before parity is seen in urology, neurosurgery, and orthopedics, and two centuries for cardiac surgery.

## Discussion

**Fig. 4:**
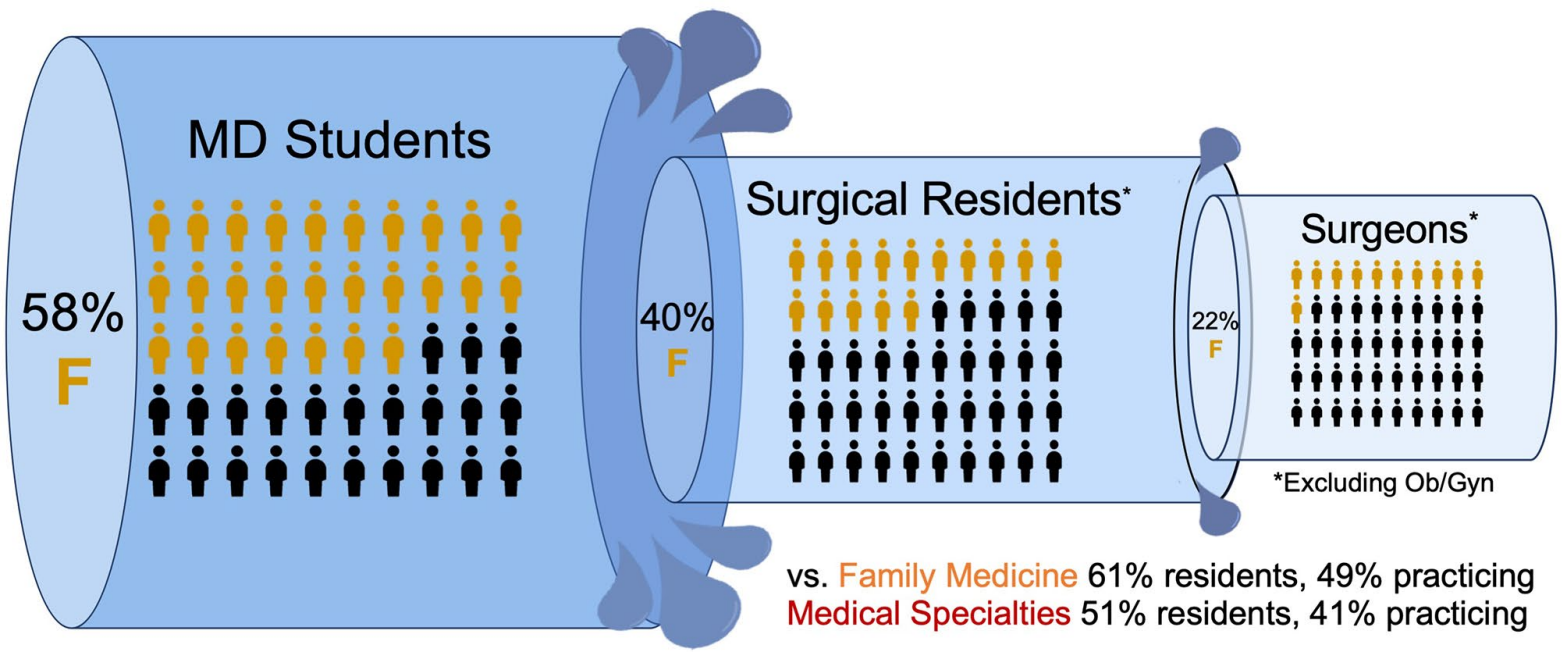
Summary of gender balance in the surgeon training pathway in Canada, illustrated as a pipeline from medical degree to surgical residency to practicing surgeon. Data from AFMC and CAPER 2020/21. *Ob/Gyn not included

Over the past three decades, medicine in Canada has seen a large shift in the gender composition of its students. While the overall proportion of females entering medical careers has increased, there are clear gender patterns in the field of training, with female students being less likely to enter most surgical specialties. While Canadian medical school classes have been majority female for decades, female students disproportionately enter Ob/Gyn, family medicine, and non-surgical specialties, resulting in ongoing gender imbalance in most surgical fields (Fig. 4).

The reasons for this are certainly multifaceted, but important to examine. One possibility that has previously been investigated is that of gender bias in the residency match process. When pooling CaRMS match statistics from 1995 to 2019, female compared to male applicants were less likely to receive a rejection to their first-choice in family medicine (OR 0.46, 95% CI 0.39–0.54) or psychiatry (OR 0.59; 95% CI 0.46–0.76) and were more likely to be rejected from all-encompassing surgery (acceptance of males OR 1.19, 95% CI 1.10–1.28) [5]. A similar analysis of 2013 to 2019 CaRMS data found smaller effects in the same direction; female students were more likely to match family medicine (RR female vs. male 1.04, 95% CI 1.03–1.05) and less likely to match a surgical discipline (RR 0.95, 95% CI 0.91-1.00), with no difference in nonsurgical disciplines [6]. There were no significant gender effects in individual surgical specialties, apart from Urology (RR 0.80, 95% CI 0.66–0.97). An earlier analysis of 1995–2004 CaRMS data also found females were more likely to match to first-ranked family medicine, psychiatry, or emergency medicine [7], with no differences for surgery [8].

Therefore, particularly in the most recent decade, the low numbers of females entering surgical specialties cannot be accounted for by bias in the residency match. The question then is why fewer female students choose to apply to surgical residencies. It is paramount that we do not assume the self-selection of a group of students out of particular specialties to be the result of a true incompatibility between their aptitudes or personal wishes and a career in those fields. While not all students, male or female, will have the desire to pursue surgery, it is important to disentangle the origin and validity of information received by medical students that may contribute to the disproportionate dissuasion of females from these fields.

While the present study is specific to the recent history of Canadian medical education, patterns of gender disparity among specialties do not appear to be nation specific. Clear patterns have been observed in the United States, Europe, Africa, the Middle East, and Asia. While surgical training programs are predominantly male, female students are more likely to enter gynecology, pediatrics, or general practice, and internal medicine is equally split [9]. Higher interest in surgery was reported by male medical students in Kenya [10], Iran [11], Japan [12], and the Netherlands [13]. Notably, female students were as likely as male students to be interested in surgery at the beginning, but not by the end of medical school [9, 14, 15]. Therefore, across multiple nations, the culture of medicine differentially shapes the specialty interests of male and female students, leading fewer female students to pursue careers in surgery.

When considering factors that may dissuade otherwise interested students from pursuing surgical specialties, a helpful concept in is the “community of practice” [16] which has been applied as a theory of medical education [17]. When the novice expresses interest in a community of practice, the group has the power to welcome them, first into a peripheral role, then through gradual delegation of responsibilities. Gender differences may exist in the early acquisition of mentors to begin this process [18]. Female medical students describe perceiving the invitation to participate in male-dominated networks as less obtainable [19]. The culture of a community of practice recreates itself in its historical image by inviting newcomers that are the right “fit”. Personality fit has been cited by students as a key factor in the decision of whether or not to pursue surgery [20, 21]. Finding belonging, and being welcomed as a newcomer, is easier for students with characteristics that align with those already in the field. In the Canadian system where objective measures of academic success are deemphasized, factors such as the identity of letter writers are heavily weighted by program directors [22], students may become especially attuned to messaging that they are not the right “fit” in a field, leading to early self-removal.

What we come to know as the culture of surgery and the personality of a surgeon are formed iteratively in a social context through the emulation of past generations. Senior residents and early-career female surgeons report exclusion from the dominant culture in departments of surgery [23]. Women in male-dominated specialties report expending mental resources on impression management to avoid the consequences of either confirming gender stereotypes (e.g., not agentic), or violating gender stereotypes and being seen as unlikable or interpersonally hostile [24, 25]. At the same time, female surgeons may distance themselves from gender issues, because to identify as having experienced discrimination or inequity would emphasize otherness and be professionally harmful [26]. As an unintended consequence of these efforts by female residents and surgeons to adapt, female students report struggling to identify with women in surgery during rotations, or to imagine themselves as successful surgeons in the future [18]. When the cost of joining a discipline is adaptation to the dominant culture, this inadvertently alienates students from potential role models.

Role models and mentorship play a key role in specialty choice. They provide students a way to imagine themselves in the “paradigmatic trajectory” of a discipline [18], and have a profound influence on professional identity formation [27]. However, female trainees are less likely compared to their male peers to have a mentor identified [28–32], and as few as 8% of current surgeons report having had a female role model or mentor [33]. This is in part the result of historically low numbers of women in surgery, and it is hopeful to see that the proportion of female surgeons who can identify a female mentor steadily increases with newer generations [33]. Greater time commitment outside the workplace for female surgeons can present an additional challenge to mentorship [32, 34]. Nonetheless, the mere presence women in positions of leadership has a positive impact on female trainees. Numbers of female students entering a specialty is directly correlated with female representation among faculty and program directors within that specialty [35–37].

Another function of role models and mentors is to dispel misconceptions by providing a forum for candid discussion. Female medical students perceive a greater conflict between a career in surgery and personal or family life than is reported by female surgeons [38]. Female surgeons report high satisfaction with their careers and degree of control over their lifestyle [23, 39]. Already in 1990, at a time only 6.5% of practicing Canadian surgeons were women, as few as 4–9% of female surgeons expressed dissatisfaction with career, marriage, health, friendships, financial status, or hobbies [40]. Despite huge advances in female representation within surgery since this time, an undercurrent of discourse that places a career in surgery at odds with family planning goals for women permeates into the present and may unduly influence specialty decisions.

While the concerns of female students may not be in proportion to the reported experience of female surgeons, concerns about role conflict are also not unfounded. As a group, female compared to male physicians allocate more time to domestic tasks and childcare responsibilities [34, 41, 42]. Additionally, female students report concerns about the difficulty of starting a family during surgical training [19]. This may reflect the ongoing stigmatization of pregnancy in surgical residency, which has been associated with career choice regret [43]. This issue clearly calls for more adequate institutional supports [43–45]. Importantly, the negative experiences of residents trickle down to students through advice discouraging a similar career path [43, 46].

As articulated 30 years ago: “If the surgical specialties are to remain competitive for the best medical school graduates, they must be able to attract female medical talent into surgical training programs” [40]. Frequent recommendations including leadership and mentorship programs, career flexibility, clearly defined parental leave policies, and continued efforts toward increased visibility, are essential and relevant to males and females alike [45, 47]. Our analysis highlights that additional attention should be paid to factors influencing the specialty preferences of students early in medical school.

A limitation of the present study is that it provides only a description of the current state. While we have speculated on reasons why fewer female medical students enter surgical subspecialties, including these frequently cited factors, as well as less commonly addressed elements of specialty culture, future research seeking the perspective of Canadian medical students is needed. Additionally, the available data is limited by the binary self-identification of gender category.

## Conclusion

Females now comprise the majority of medical graduates, albeit, a minority of those entering surgical fields. The topic of choosing a medical specialty should be formally addressed to inform this decision making, rather than leaving the influencing power to the hidden curriculum and the unaddressed historical biases within it. The goal is to enable students to truly *choose* a career path, unconstrained by echoes of past prejudice. While it is essential for trainees to see female leaders, meaningful mentorship can come from a variety of sources, with engagement being more important to students than gender concordance [19]. Anyone involved in medical student education has the decision to unreflectively uphold the current culture, or to actively welcome and encourage a diversity of students into their field. The future of medicine depends in a very real sense on who, among the talented young students, is given the chance to feel that they “fit”.

## Data Availability

The datasets used during the current study are freely available from the respective sources (AFMC, CAPER, and CIHI). Those generated by the present study are available from the corresponding author (L.P.) on reasonable request.
